# Estimated Impact of Nirsevimab on the Incidence of Respiratory Syncytial Virus Infections Requiring Hospital Admission in Children < 1 Year, Weeks 40, 2023, to 8, 2024, Spain

**DOI:** 10.1111/irv.13294

**Published:** 2024-05-08

**Authors:** Clara Mazagatos, Jacobo Mendioroz, Mercedes Belén Rumayor, Virtudes Gallardo García, Virginia Álvarez Río, Ana Delia Cebollada Gracia, Noa Batalla Rebollo, María Isabel Barranco Boada, Olaia Pérez‐Martínez, Ana Sofía Lameiras Azevedo, Nieves López González‐Coviella, Daniel Castrillejo, Ana Fernández Ibáñez, Jaume Giménez Duran, Cristina Ramírez Córcoles, Violeta Ramos Marín, Amparo Larrauri, Susana Monge, The SARI Sentinel Surveillance RSV Study Group, Luca Basile, Nicola Lorusso, María Teresa Otero‐Barrós, Marcos Lozano, Luis García Comas, María del Carmen Pacheco Martínez, Cristina Rodríguez Martí, Juan Antonio Linares Dópido, Alonso Sánchez‐Migallon Naranjo, Miriam Lopez Torrijos, Eva Rivas Wagner, Luisa F. Hermoso Castro, Marta Huerta Huerta, Jorge Reina Prieto, Ana Gómez Juárez, Aranzazu Ruiz Nuñez, Fernando González Carril, Liher Imaz Goienetxea, Ana Carmen Ibáñez Pérez, Belén Berradre Saenz, Tania Puma Olguin, Gloria Pérez Gimeno, Silvia Galindo Carretero, Lorena Vega Piris

**Affiliations:** ^1^ National Centre of Epidemiology Institute of Health Carlos III Madrid Spain; ^2^ CIBER Epidemiology and Public Health Madrid Spain; ^3^ Sub‐direcció General de Vigilància i Resposta a Emergències de Salut Pública, Departament de Salut Generalitat de Catalunya Barcelona Spain; ^4^ Área de Enfermedades Transmisibles, Subdirección General de Vigilancia en Salud Pública Madrid Spain; ^5^ Servicio de Vigilancia y Salud Laboral Dirección General de Salud Pública y Ordenación Farmacéutica, Consejería de Salud y Consumo Andalucía Spain; ^6^ Servicio de Epidemiología, Consejería de Sanidad, Dirección General de Salud Pública Junta de Castilla y León Valladolid Spain; ^7^ Sección de Vigilancia Epidemiológica, Dirección General de Salud Pública Zaragoza Aragón Spain; ^8^ Subdirección de Epidemiología de la Dirección General de Salud Pública Servicio Extremeño de Salud Mérida Spain; ^9^ Servicio de Epidemiología (Sección Vigilancia Epidemiológica) Consejería de Salud‐Región de Murcia Murcia Spain; ^10^ Servizo de Epidemioloxía, Dirección Xeral de Saúde Pública, Consellería de Sanidade Xunta de Galicia Santiago Spain; ^11^ Subdirecció General d'Epidemiologia i Vigilància de la Salut, Direcció General de Salut Pública Generalitat Valenciana Valencia Spain; ^12^ Servicio de Epidemiología de la Dirección General de Salud Pública, Servicio Canario de la Salud Tenerife Spain; ^13^ Vigilancia Epidemiológica, Consejería de Políticas Sociales y Salud Pública de Melilla Dirección General de Salud Pública Melilla Spain; ^14^ Dirección General de Salud Pública y Atención a la Salud Mental Consejería de Sanidad, Principado de Asturias Oviedo Spain; ^15^ Servicio de Epidemiología, Consellería de Salut Gobierno de las Islas Baleares Palma Spain; ^16^ Instituto de Investigación Sanitaria Illes Balears (IdISBa) Palma Spain; ^17^ Sección de Epidemiología Delegación Provincial de Sanidad de Albacete Albacete Spain; ^18^ Servicio de Epidemiología Consejería de Sanidad y Servicios Sociales de Ceuta Ceuta Spain; ^19^ CIBER Infectious Diseases Madrid Spain

**Keywords:** burden, impact, respiratory infections, respiratory syncitial virus, SARI, surveillance

## Abstract

**Background:**

Data from the sentinel surveillance system of severe acute respiratory infections in Spain were used to estimate the impact of administration of nirsevimab to children born from 1 April 2023 onwards.

**Methods:**

Estimated RSV hospitalisations in < 1‐year‐olds during weeks 40, 2023, to 8, 2024, were compared to the number that would be expected after accounting for the background change in RSV circulation in the 2023/24 season, compared to 2022/23.

**Results:**

We estimated 9364–9875 RSV hospitalisations less than expected, corresponding to a 74%–75% reduction.

## 1 Background

It has been estimated that 1.8% of newborns in Europe will be hospitalized due a respiratory syncytial virus (RSV) infection during their first year of life [1]. Nirsevimab (Beyfortus) is a monoclonal antibody approved by the European Medicines Agency in October 2022 to prevent serious lower respiratory tract disease caused by RSV in infants during their first RSV season [2, 3]. Spain is one of the four countries globally, together with Luxembourg, France and the United States of America, that has recommended preventive administration of nirsevimab to previously health children for the 2023/24 season.

Starting on 1 October 2023, public health authorities in Spain recommended administration of nirsevimab, free of charge, to all infants born on or after 1 April 2023 [4, 5] either administered as soon as possible after birth (normally within the first 48 h of life) or as catch‐up immunisation for those born before the recommendation was in place. Nirsevimab was also recommended to premature (< 35 weeks gestational age) children < 12 months and to children with other risk conditions < 24 months of age. For the differences by autonomous communities (ACs), see Table S1. Acceptance has been being very good. According to the Spanish Ministry of Health [6], mean coverage in newborns, catch‐up vaccination, premature children or other children at risk has been, respectively, 92% (86%–97%, by ACs), 87% (45%–9%), 85% (43%–100%) and 94% (75%–100%).

Our objective is to estimate the impact of this recommendation in terms of prevented cases of RSV infections requiring hospital admission in children < 1 year, by comparing estimated RSV hospitalizations in < 1‐year‐olds during epidemiological weeks 40, 2023, to 8, 2024, to what would have been expected in the same period in the absence of nirsevimab administration.

## 2 Materials and Methods

### 2.1 Estimation of RSV Hospitalisations in < 1‐Year‐Olds

We analysed data from the Surveillance System of Acute Respiratory Infections in Spain (SiVIRA), an integrated system that monitors acute respiratory infections (ARI) in primary care and Severe ARI (SARI) in hospitals. The impact assessment focused on SARI data, though we also analysed ARI to provide the wider context of RSV circulation (Figures S1 and S3 and Tables S2 and S3). Between 24 and 27 sentinel hospitals in 15 of 19 ACs in Spain provided weekly aggregated numbers (stratified by pre‐defined age groups) of patients admitted with SARI, defined as ARI (cough, shortness of breath, coryza or sore throat and clinical judgement of an infection) with acute onset in the last 10 days and hospitalized for ≥ 24 h. In contrast to WHO and ECDC definitions [7], fever was not a requirement. Hospitals also reported the catchment population size by age group, allowing the calculation of age‐specific SARI incidence rates (Figure S2).

**FIGURE 1:**
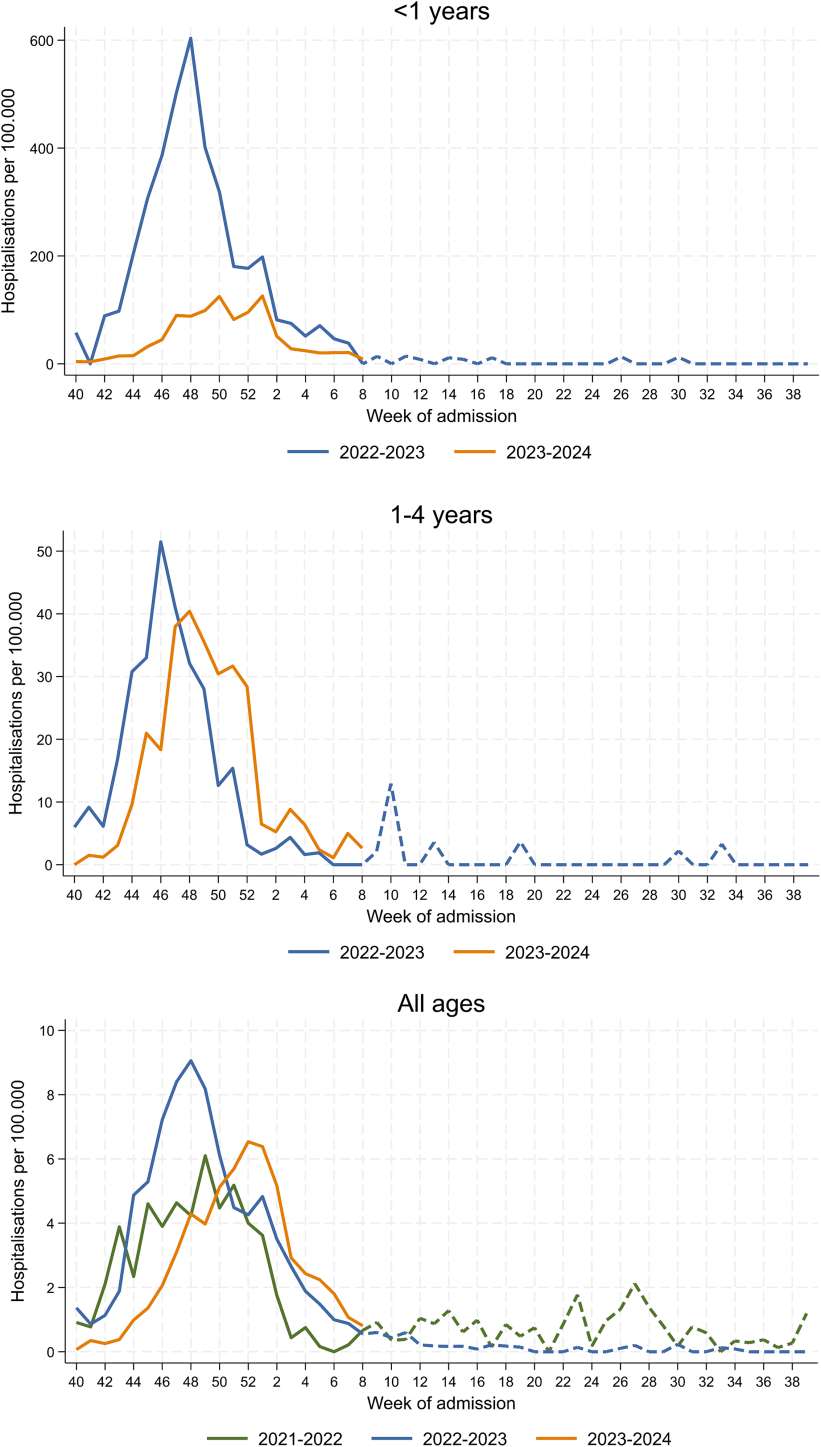
Weekly respiratory syncytial virus (RSV) *proxy* hospitalisation rates, by age group and respiratory season (from week 40, to week 39 of the next year), sentinel SARI surveillance system in Spain. The *proxy* is obtained by multiplying the Severe Acute Respiratory Infection (SARI) syndromic hospitalisation rates by the proportion of laboratory tests positive for RSV among those with SARI and systematically tested. Stratification for groups < 1 year or 1–4 years is only available for season 2022/23 onwards.

Further, a systematic sample of weekly SARI cases is selected (normally, all admissions on predefined weekdays) for in‐depth collection of clinical, epidemiological and virological data, including systematic swab and PCR testing for influenza, SARS‐CoV‐2 and RSV. Positivity rates among those systematically tested (Table S2) are then applied to syndromic rates to obtain a *proxy* of pathogen‐specific SARI hospitalisation rates [8]. Using this approach, we computed weekly RSV‐specific *proxy* hospitalization rates (hereafter, RSV hospitalisation rates; Figure 1).

Finally, we estimated the corresponding number of RSV hospitalisations by age group (Table S3) applying the RSV hospitalisation rate to the population size by age group, in Spain. The estimated number of RSV hospitalisations during weeks 40, 2023, to 8, 2024, were taken as observed (O, Table 1).

**TABLE 1:** Estimated number of respiratory syncytial virus (RSV) hospitalisations in Spain, by age group.

	Observed (O), weeks 40, 2022, to 8, 2023	Observed (O), weeks 40, 2023, to 8, 2024	Scaling factor (2023/24 vs. 2022/23)	Model	Expected (E) in <1 year, 2023/24^a^	Incidence ratio (O/E) in <1 year	Averted hospitalisations (E − O) in <1 year
<1 year	13,120	3357	NA	Crude	13,120	0.26	9763
1–4 years	4494	4357	0.97	A	12,721	0.26	9364
1–110 years	19,688	19,856	1.01	B	13,233	0.25	9875

*Note:* Number of cases between weeks 40 to 8 are estimated for seasons 2022/23 and 2023/24^a^ and referred to as observed (O). Expected cases (E) in < 1‐year‐olds in 2023/24 are directly obtained from the observed cases in 2022/23 in < 1‐year‐olds in the ‘crude’ analysis or by applying to these the scaling factor from 1‐ to 4‐year‐olds (Model A) or from 1‐ to 110‐year‐olds (Model B). Incidence ratios and number of averted RSV hospitalisations are then computed.

Abbreviation: NA, not applicable.

^a^ The RSV *proxy* hospitalisation rates are applied to the population size by age group and autonomous community; data are aggregated across autonomous communities for the totals by age groups shown in the table. Numbers are not reproducible by hand‐calculation due to the inclusion of multiple decimal positions.

### 2.2 Estimation of Expected RSV Hospitalisations in < 1‐Year‐Olds

We encountered several challenges to estimate the number of hospitalisations in children < 1 year, in weeks 40/2023 to 8/2024, that would have been expected in the absence of nirsevimab administration. First, although RSV surveillance within SiVIRA has been in place since 2021/22, the breakdown for age < 1 year was only included in SARI surveillance in the 2022/23 season [9]. Thus, only one previous season was available as reference for the expected number of cases. Second, after the COVID‐19 pandemic lockdown in 2020, RSV circulation has had altered seasonality and higher intensity, possibly related to very low circulation and accumulation of susceptibles among those born from 2020 onwards [10, 11, 12, 13]. The unstable circulation adds uncertainty in the extrapolation of the 2022/23 season to the current one. Indeed, ARI and SARI rates from SiVIRA, both general and RSV specific, have been very similar overall in the current 2023/24 season compared to the previous one (Figures 1 and S1–S3).

We first directly took the number of RSV hospitalisations observed in the < 1‐year‐olds in weeks 40/2022 to 8/2023 as the expected number in the equivalent period in the current 2023/24 season (‘crude’ analysis). However, to estimate the expected cases accounting for the possible different seasonality and intensity of RSV circulation, we multiplied the observed RSV hospitalisations in the current 2023/24 season by a scaling factor, equal to the ratio between RSV hospitalisations in the current 2023/24 season and RSV hospitalisations in the same weeks of the previous season. We computed this scaling factor for age groups not included in the generalised recommendation for nirsevimab (1‐ to 4‐year‐olds and 1‐ to 110‐year‐olds), as alternative valid approaches.

**FIGURE 2:**
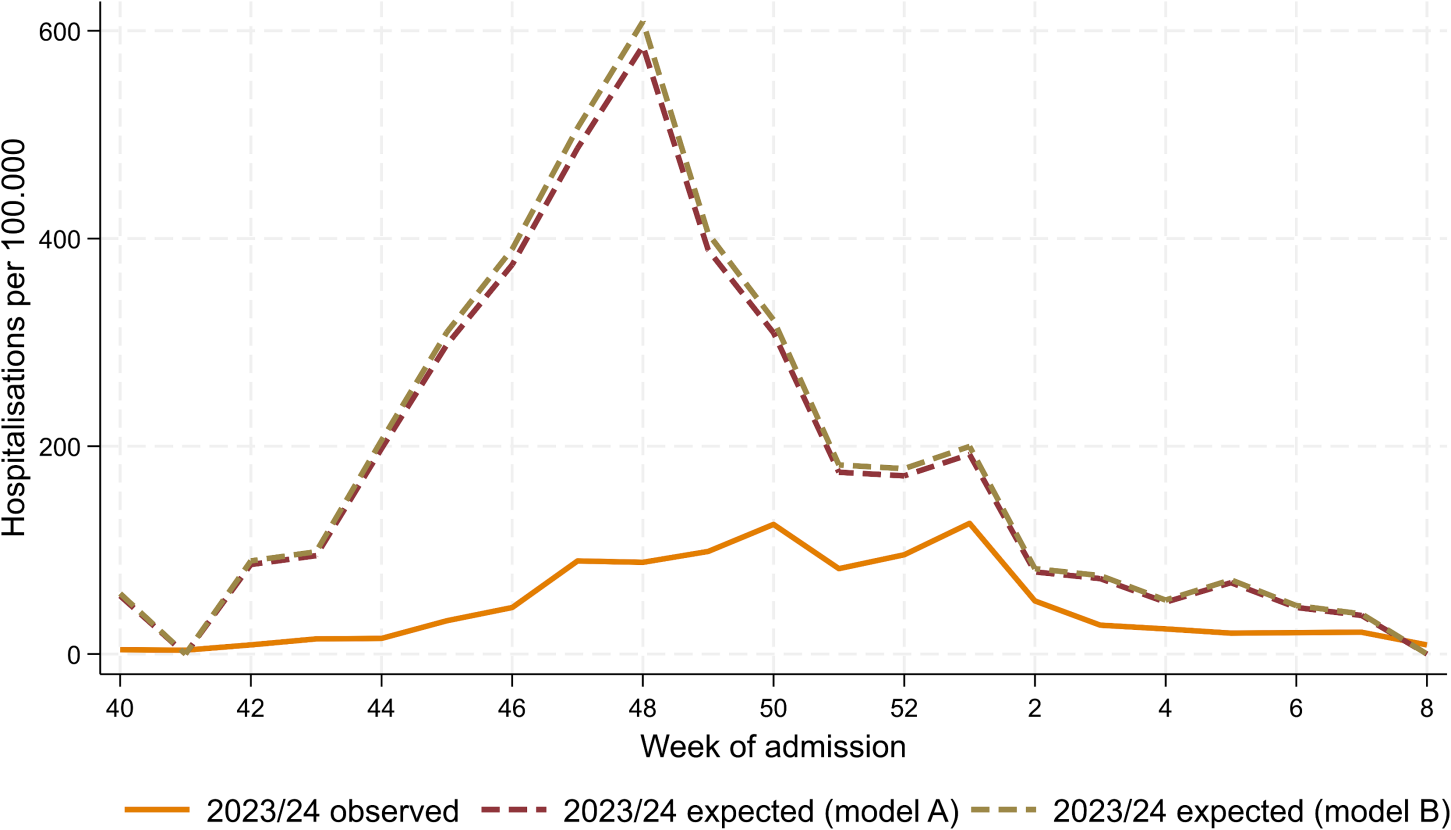
Estimated number of observed respiratory syncytial virus (RSV) hospitalisations in < 1‐year‐olds in Spain, weeks 40/2023–8/2024. Expected cases are obtained by applying to the observed cases from the equivalent weeks in 2022/23 a scaling factor (see Table 1) in 1‐ to 4‐year‐olds (2023/24 expected [Model A]) or 1‐ to 110‐year‐olds (2023/24 expected [Model B]). The RSV *proxy* hospitalisation rates each week are applied to the population size by age group and autonomous community; data are aggregated across autonomous communities for the number of weekly cases in Spain, referred to as observed.

Compared to the 2022/23 season, RSV hospitalisations in the 2023/24 season were very similar in the population 1 year of age or older, but considerably lower in the < 1‐year‐olds (Table 1). Results of estimated expected cases in < 1‐year‐olds are shown in Table 1 and Figure 2.

### 2.3 Estimated Impact of Nirsevimab: Comparing Observed and Expected

The observed (O) number of RSV hospitalisations was compared to the expected (E) in children < 1 year of age for weeks 40/2023 to 8/2024. We compared them using (i) ratios (O/E), to derive the relative reduction in RSV hospitalisations attributable to nirsevimab, and (ii) differences (E − O), to derive the number of averted RSV hospitalisations attributable to nirsevimab (Table 1).

We estimated that, during weeks 40/2023 to 8/2024, the administration of nirsevimab reduced RSV hospitalisations in < 1‐year‐olds by between 74% and 75%, depending on the scaling factor used. This resulted in between 9364 and 9875 averted RSV hospitalisations in this group and period.

## 3 Discussion

Our results indicate an important reduction in the incidence of RSV infections requiring hospital admission in children < 1 year old during weeks 40/2023 to 8/2024, compared to what would have been expected with reference to the same period in the 2022/23 season.

Of note, not all children admitted < 1 year of age were targeted for nirsevimab, though a majority of them was (those born on or after 1 April 2023). A finer definition of the cohort targeted for nirsevimab was not possible with our data. Likewise, a small fraction of children with at‐risk conditions over 1 year of age, who was previously targeted for palivizumab (Synagis), was recommended nirsevimab for this 2023/24 season [5]. The change between seasons among population 1 year or older attributable to nirsevimab is therefore considered negligible. The similarity of all estimations independently of the reference group used gives our results more credibility.

As a further limitation, we acknowledge great uncertainty in the estimation of expected cases, especially in the context of the unstable circulation of respiratory viruses after the COVID‐19 pandemic [10, 11, 12, 13]. However, the SARI case definition and the systematic testing criteria in SiVIRA surveillance [14] did not change throughout the study period. Similar studies based on data sources with longer time‐series, such as hospital discharge records [12], could further explore the implications of using as reference the 2022/23 season.

Our study has estimated a 74%–75% relative reduction in the risk of RSV hospitalisation, highly comparable to the clinical efficacy shown in pivotal randomised control trials, of 77% [3, 15]. Our estimates were expected to be lower, since not all children < 1 year old were immunised. However, they could be also higher due to certain indirect impact in the nonimmunised population, although nirsevimab does not prevent the development of an immune response to RSV [16] and is not expected to affect RSV transmission. Few other estimates are available from real‐world data. In three regions in Spain [17], unadjusted effectiveness was 69%–97% using a test‐negative case–control design or the screening method. In Luxembourg [18], a 69% decrease in RSV hospitalisations in infants under 6 months old was also observed, along a change in the age structure of children admitted to hospital due to RSV, indicating a high impact of nirsevimab.

In summary, our results based on data from SARI sentinel surveillance in Spain provide additional evidence on the benefits of nirsevimab administration to prevent RSV hospitalisations in children < 1 year of age in real‐world conditions. Moreover, we provide estimates of its impact in terms of averted RSV hospitalisations, something key for future cost‐effectiveness studies. Our findings can help inform recommendations on the use of nirsevimab at a population level globally, and have already informed the new recommendations in Spain for the upcoming 2024/25 respiratory season [6]. However, it is of outmost importance that further studies with individual‐level data can confirm and more accurately quantify the effectiveness and impact of nirsevimab, particularly in the near‐future landscape of different alternatives for the prevention of severe RSV infection in children [19].

## Author Contributions

**Clara Mazagatos:** conceptualization, data curation, formal analysis, investigation, methodology, visualization, writing–original draft, writing–review and editing. **Jacobo Mendioroz:** data curation, investigation, writing–review and editing. **Mercedes Belén Rumayor:** data curation, investigation, writing–review and editing. **Virtudes Gallardo García:** data curation, investigation, writing–review and editing. **Virginia Álvarez Río:** data curation, investigation, writing–review and editing. **Ana Delia Cebollada Gracia:** data curation, investigation, writing–review and editing. **Noa Batalla Rebollo:** data curation, investigation, writing–review and editing. **María Isabel Barranco Boada:** data curation, investigation, writing–review and editing. **Olaia Pérez‐Martínez:** data curation, investigation, writing–review and editing. **Ana Sofía Lameiras Azevedo:** data curation, investigation, writing–review and editing. **Nieves López González‐Coviella:** data curation, investigation, writing–review and editing. **Daniel Castrillejo:** data curation, investigation; writing–review and editing. **Ana Fernández Ibáñez:** data curation, investigation, writing–review and editing. **Jaume Giménez Duran:** data curation, investigation, writing–review and editing. **Cristina Ramírez Córcoles:** data curation, investigation, writing–review and editing. **Violeta Ramos Marín:** data curation, investigation, writing–review and editing. **Amparo Larrauri:** conceptualization, investigation, methodology, supervision, writing–original draft, writing–review and editing. **Susana Monge:** conceptualization, investigation, methodology, supervision, writing–original draft, writing–review and editing.

## Ethics Statement

All data used for this study were collected as routine surveillance, and informed consent or official ethical approval was not required, as regulated by Royal Decree 2210/1995 of December 28 provided by the Ministry of Health and Consumer Affairs. Although individual informed consent was not required, all data were pseudoanonymised to protect patient privacy and confidentiality.

## Conflicts of Interest

The authors declare no conflicts of interest.

### Peer Review

The peer review history for this article is available at https://www.webofscience.com/api/gateway/wos/peer‐review/10.1111/irv.13294.

## Supporting information


**Table S1.** Minimum date of birth for eligibility for immunisation with nirsevimab (children born on or after the given dates were eligible, under different indications) in the 19 autonomous communities.
**Figure S1.** Weekly incidence of Acute Respiratory infections (ARI) attended in primary care, by age group, SiVIRA, seasons 2021–22 to 2023–24.
**Figure S2.** Weekly incidence of Severe Acute Respiratory infections (SARI) in hospitals, by age group, SiVIRA, seasons 2021–22 to 2023–24.
**Table S2.** Number of ARI and SARI patients tested for RSV and number and proportion of RSV positives, by age group and season, SiVIRA, seasons 2022–23 and 2023–24.
**Figure S3.** Weekly incidence of RSV infections* in primary care by age group, SiVIRA, seasons 2021–22 to 2023–24.
**Table S3.** Age distribution of confirmed RSV infections in primary care and RSV hospitalisations, SiVIRA, seasons 2022–23 and 2023–24.

## Data Availability

Data access policy within the National Epidemiological Surveillance Network (RENAVE) is similar to that of other Public Health Agencies, such as the European Centre for Disease Control. The National Centre of Epidemiology has the mandate to collect, analyse and disseminate surveillance data on infectious diseases in Spain. There is no direct access to the RENAVE database, but data used for this study are available upon request to the corresponding author.
