# Correction to ‘Estimated Impact of Nirsevimab on the Incidence of Respiratory Syncytial Virus Infections Requiring Hospital Admission in Children < 1 Year, Weeks 40, 2023, to 8, 2024, Spain’

**DOI:** 10.1111/irv.70043

**Published:** 2024-11-12

Mazagatos, C., Mendioroz, J., Rumayor, M., Gallardo García, V., Álvarez Río, V., Cebollada Gracia, A., Batalla Rebollo, N., Barranco Boada, M., Pérez‐Martínez, O., Lameiras Azevedo, A., López González‐Coviella, N., Castrillejo, D., Fernández Ibáñez, A., Giménez Duran, J., Ramírez Córcoles, C., Ramos Marín, V., Larrauri, A., Monge, S. and (2024), Estimated Impact of Nirsevimab on the Incidence of Respiratory Syncytial Virus Infections Requiring Hospital Admission in Children < 1 Year, Weeks 40, 2023, to 8, 2024, Spain. *Influenza Other Respi Viruses*, 18: e13294. https://doi.org/10.1111/irv.13294

In the article, there was an error in the denominators reported by one of the regions contributing to the national SiVIRA surveillance, affecting both the primary care and hospital catchment populations. This error affects the calculation of national SARI hospitalisation rates for the 2022–2023 and 2023–2024 seasons, as well as all subsequent estimates of RSV‐specific proxy hospitalisation rates and the observed and expected number of RSV hospitalisations. Although Figures 1 and 2 and the published estimates in Table 1 have changed, this correction does not change the overall study conclusions.

**FIGURE 1:**
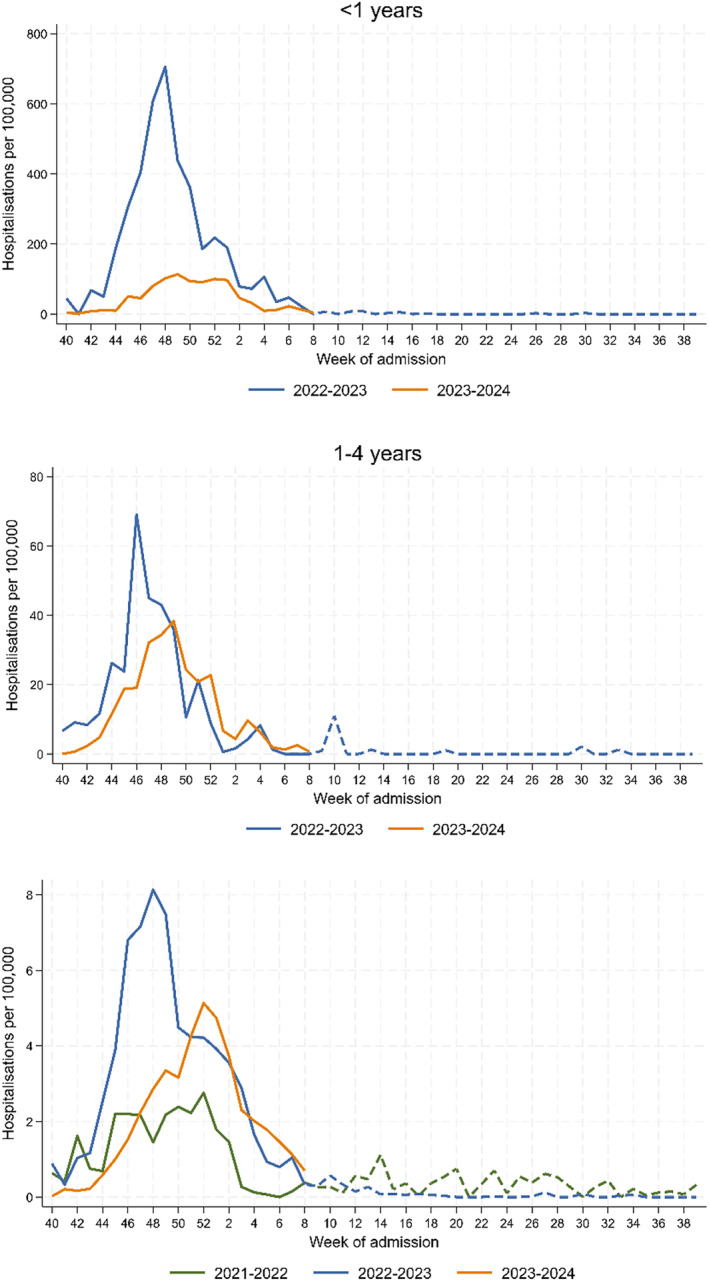
Weekly respiratory syncytial virus (RSV) *proxy* hospitalisation rates, by age group and respiratory season (from week 40 to week 39 of the next year), sentinel SARI surveillance system in Spain. The *proxy* is obtained by multiplying the severe acute respiratory infection (SARI) syndromic hospitalisation rates by the proportion of laboratory tests positive for RSV among those with SARI and systematically tested. Stratification for groups < 1 year or 1–4 years is only available for season 2022/2023 onwards.

**FIGURE 2:**
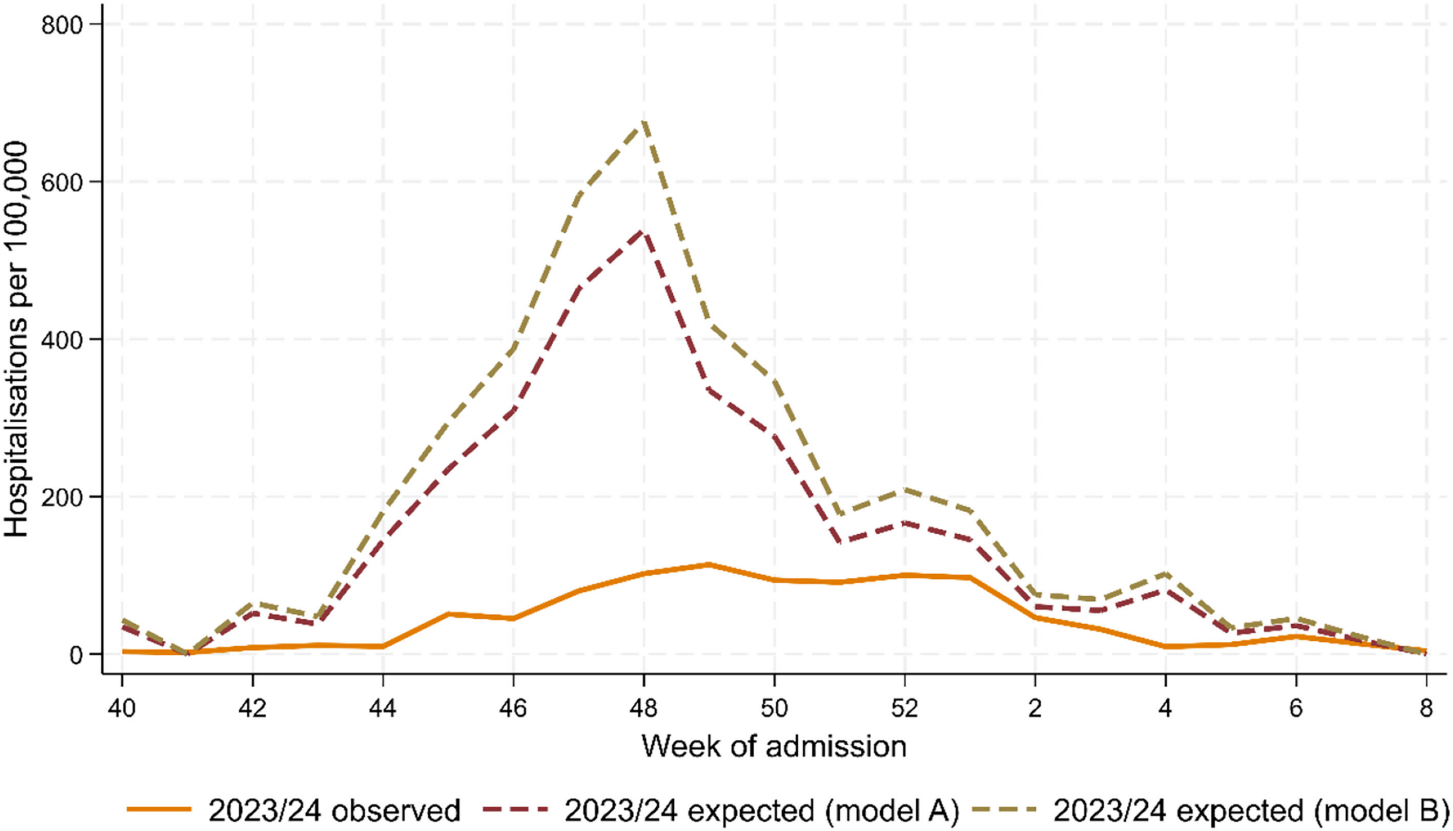
Estimated number of observed respiratory syncytial virus (RSV) hospitalisations in < 1‐year‐olds in Spain, weeks 40/2023–8/2024. Expected cases are obtained by applying to the observed cases from the equivalent weeks in 2022/2023 a scaling factor (see Table 1) in 1‐ to 4‐year‐olds (2023/2024 expected [Model A]) or 1‐ to 110‐year‐olds (2023/2024 expected [Model B]). The RSV *proxy* hospitalisation rates each week are applied to the population size by age group and autonomous community; data are aggregated across autonomous communities for the number of weekly cases in Spain, referred to as observed.

**TABLE 1:** Estimated number of respiratory syncytial virus (RSV) hospitalisations in Spain, by age group.

	Observed (O), weeks 40, 2022, to 8, 2023	Observed (O), weeks 40, 2023, to 8, 2024	Scaling factor (2023/2024 vs. 2022/2023)	Model	Expected (E) in < 1 years, 2023/2024^a^	Incidence ratio (O/E) in < 1 years	Averted hospitalisations (E–O) in < 1 years
< 1 year	13.916	3123	NA	Crude	13.916	0.22	10.793
1–4 years	5016	3833	0.76	A	10.633	0.29	7.510
1–110 years	18.116	17.360	0.96	B	13.335	0.23	10.213

*Note:* Number of cases between weeks 40 to 8 are estimated for seasons 2022/2023 and 2023/2024^a^ and referred to as observed (O). Expected cases (E) in < 1‐year‐olds in 2023/2024 are directly obtained from the observed cases in 2022/2023 in < 1‐year‐olds in the ‘crude’ analysis or by applying to these the scaling factor from 1‐ to 4‐year‐olds (Model A) or from 1‐ to 110‐year‐olds (Model B). Incidence ratios and number of averted RSV hospitalisations are then computed.

Abbreviation: NA, not applicable.

^a^ The RSV *proxy* hospitalisation rates are applied to the population size by age‐group and autonomous community; data are aggregated across autonomous communities for the totals by age‐groups shown in the table. Numbers are not reproducible by hand‐calculation due to the inclusion of multiple decimal positions.

The corrected Table 1 is as follows:

The corrected Figures 1 and 2 are as follows:

The text should be corrected every time these estimates are mentioned: In Section 2.3, ‘Estimated Impact of Nirsevimab: Comparing Observed and Expected’, the text in the second paragraph ‘We estimated that, during weeks 40/2023 to 8/2024, the administration of nirsevimab reduced RSV hospitalisations in < 1‐year‐olds by between 74% and 75%, depending on the scaling factor used. This resulted in between 9364 and 9875 averted RSV hospitalisations in this group and period’ was incorrect and should read as ‘We estimated that, during weeks 40/2023 to 8/2024, the administration of nirsevimab reduced RSV hospitalisations in < 1‐year‐olds by between 71% and 77%, depending on the scaling factor used. This resulted in between 7510 and 10,213 averted RSV hospitalisations in this group and period’.

In the discussion section, fourth paragraph, the text ‘Our study has estimated a 74%–75% relative reduction in the risk of RSV hospitalisation …’ was incorrect and should read as ‘Our study has estimated a 71%–77% relative reduction in the risk of RSV hospitalisation …’.

The supplement of this article has also been amended. The corrected Figures S1, S2 and S3 are as follows:

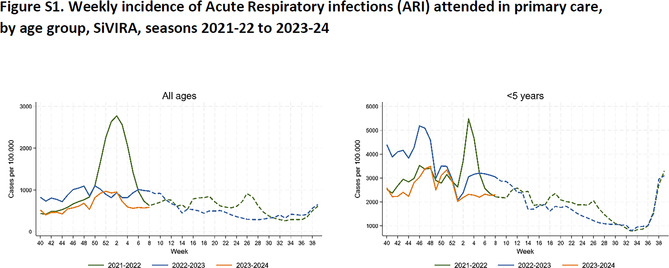

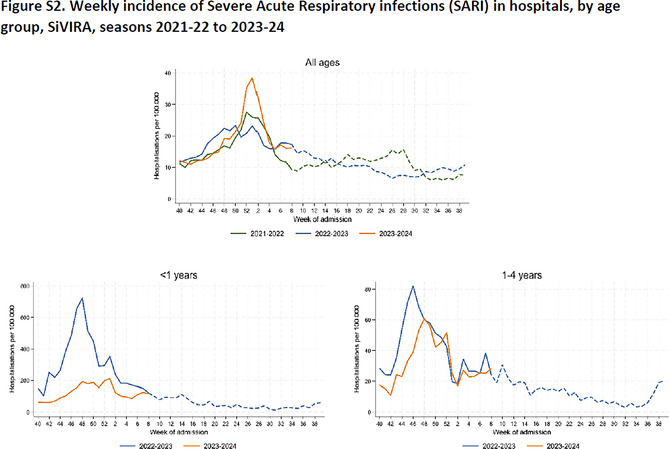

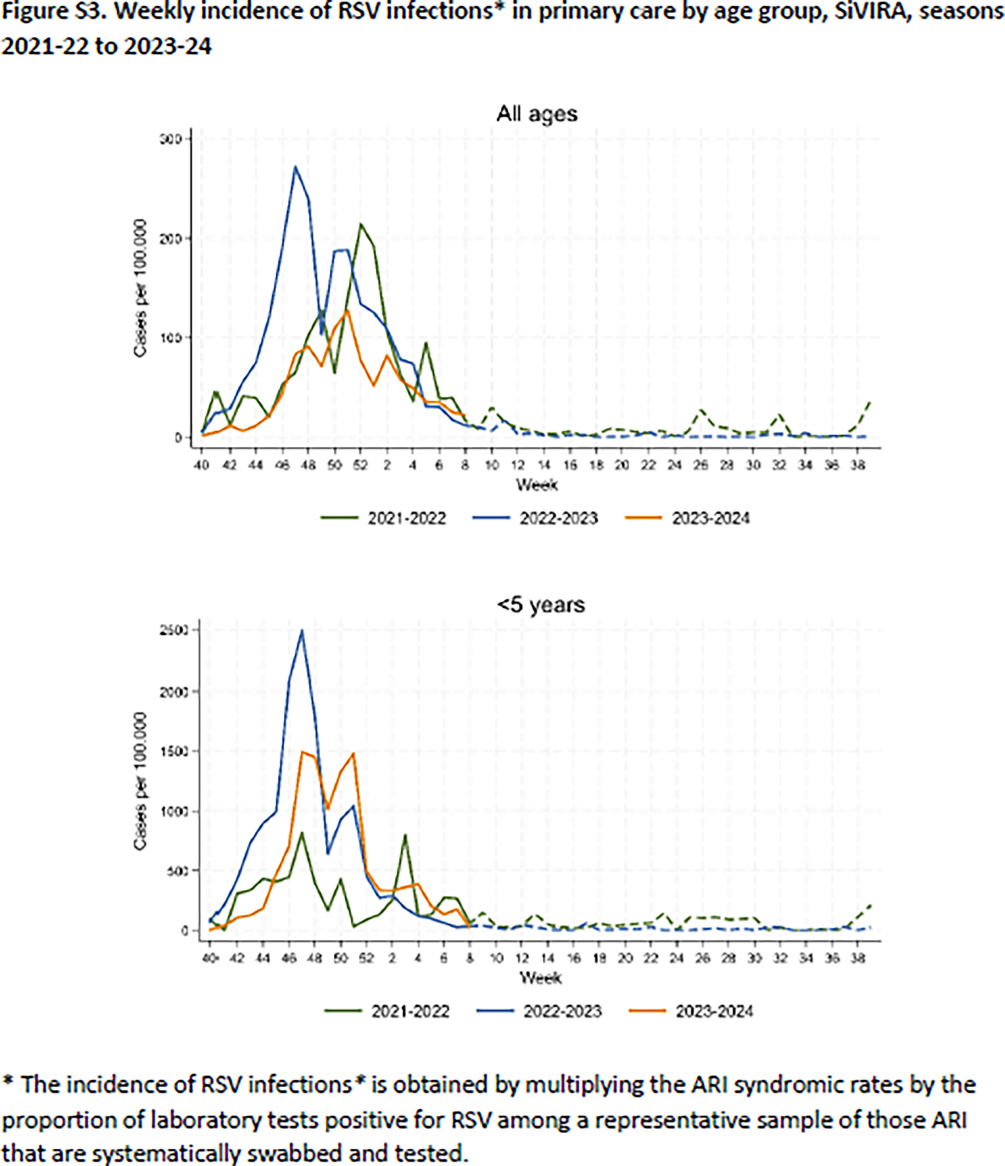

And corrected Table S3 is as follows:

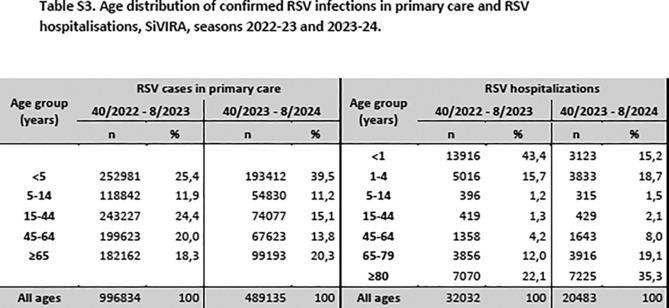

We apologise for this error.

