# Supplementary material for: Estimated Impact of Nirsevimab on the Incidence of Respiratory Syncytial Virus Infections Requiring Hospital Admission in Children < 1 Year, Weeks 40, 2023, to 8, 2024, Spain
Source: Influenza Other Respir Viruses. 2024 May 8;18(5):e13294. doi: 10.1111/irv.13294 (PMC11077568; doi:10.1111/irv.13294)
Supplement: Supplementary file 1 — Table S1. Minimum date of birth for eligibility for immunisation with nirsevimab (children born on or after the given dates were eligible, under different indications) in the 19 autonomous communities. Figure S1. Weekly incidence of Acute Respiratory infections (ARI) attended in primary care, by age group, SiVIRA, seasons 2021–22 to 2023–24. Figure S2. Weekly incidence of Severe Acute Respiratory infections (SARI) in hospitals, by age group, SiVIRA, seasons 2021–22 to 2023–24. Table S2. Number of ARI and SARI patients tested for RSV and number and proportion of RSV positives, by age group and season, SiVIRA, seasons 2022–23 and 2023–24. Figure S3. Weekly incidence of RSV infections* in primary care by age group, SiVIRA, seasons 2021–22 to 2023–24. Table S3. Age distribution of confirmed RSV infections in primary care and RSV hospitalisations, SiVIRA, seasons 2022–23 and 2023–24. [file IRV-18-e13294-s001.docx]

**Estimated impact of Nirsevimab on the incidence of Respiratory Syncytial Virus infections requiring hospital admission in children <1 year, weeks 40, 2023, to 8, 2024, Spain**

**Supplementary material**

[1. Official recommendations for administration of nirsevimab in the 19 Spanish Autonomous Communities 2](#_Toc153987916)

[2. Results from syndromic surveillance of Acute Respiratory Infections (ARI) and Severe ARI (SARI) in Spain, seasons 2022-23 and 2023-24 3](#_Toc153987917)

[3. Respiratory Syncytial Virus (RSV) positivity and incidence of RSV infections medically attended in primary healthcare in Spain, seasons 2022-23 and 2023-24 4](#_Toc153987918)

## Official recommendations for administration of nirsevimab in the 19 Spanish Autonomous Communities

**Table S1. Minimum date of birth for eligibility for immunisation with nirsevimab (children born on or after the given dates were eligible, under different indications) in the 19 autonomous communities**

| **Autonomous community** | **Immunisation of newborns*** | **Catch-up immunisation** | **Children at increased risk (exc.premature)**** | **Premature children***** |
| --- | --- | --- | --- | --- |
| **Andalusia** | 01/10/2023 | 01/04/2023 | 01/10/2021 | 01/10/2022 |
| **Aragon** | 01/10/2023 | 01/04/2023 | 01/10/2021 | 01/10/2022 |
| **Asturias** | 02/10/2023 | 01/04/2023 | 01/10/2021 | 01/10/2022 |
| **Balearic Islands** | 20/11/2023 | 01/04/2023 | 01/10/2021 | 01/10/2022 |
| **Canary Islands** | 05/10/2023 | 01/04/2023 | 01/10/2021 | 01/10/2022 |
| **Cantabria (not participating in SARI surveillance)** | 01/10/2023 | 01/04/2023 | 01/10/2021 | 01/10/2022 |
| **Castille - La Mancha** | 02/10/2023 | 01/04/2023 | 01/10/2021 | 01/10/2022 |
| **Castille and Leon** | 01/10/2023 | 01/03/2023 | 01/10/2021 | 01/10/2022 |
| **Catalonia** | 02/10/2023 | 01/04/2023 | 01/10/2021 | 01/10/2022 |
| **Valencian Community** | 01/10/2023 | 01/04/2023 | 01/10/2021 | 01/10/2022 |
| **Extremadura** | 30/10/2023 | 01/05/2023 | 01/10/2021 | 01/10/2022 |
| **Galicia** | 25/09/2023 | 01/04/2023 | 01/10/2021 | 01/10/2022 |
| **Madrid** | 01/10/2023 | 01/04/2023 | 01/10/2021 | 01/10/2022 |
| **Murcia** | 25/09/2023 | 01/04/2023 | 01/10/2021 | 01/10/2022 |
| **Navarre****(not participating in SARI surveillance)** | 01/10/2023 | Not recommended | 01/10/2021 | 01/10/2022 |
| **Basque Country (only syndromic SARI surveillance)** | 13/11/2023 | 01/07/2023 | 01/10/2021 | 01/10/2022 |
| **La Rioja (only syndromic SARI surveillance)** | 01/10/2023 | 01/04/2023 | 01/10/2021 | 01/10/2022 |
| **Ceuta** | 06/10/2023 | 01/04/2023 | 01/10/2021 | 01/10/2022 |
| **Melilla** | 17/10/2023 | 01/04/2023 | 01/10/2021 | 01/10/2022 |

* The recommendation is to administrate nirsevimab as soon as possible after birth, normally in the first 48 hours (in the maternity or hospital), or alternatively in the first pediatric consultation, happening generally before 10 days of life.

** Children with increased risk of severe RSV infection were previously recommended palivizumab and this season are targeted for nirsevimab instead. Conditions defining high risk are the following: Bronchopulmonary dysplasia, congenital heart disease with significant hemodynamic alteration (those receiving treatment for congestive heart failure, those with moderate or severe pulmonary hypertension and children with cyanotic heart disease), congenital metabolic disorders, Down syndrome, cystic fibrosis, diseases neuromuscular lung diseases or malformations of the airways that hinder the ability to eliminate secretions from the upper respiratory tract, severe immunosuppression due to oncohematological processes, primary immunodeficiencies (especially combined and congenital agammaglobulinemia), confirmed HIV infection and under continuous treatment with immunosuppressants, infants in palliative care

*** Born with <35 weeks gestational age

**** Campaign finishing on 31 December with the possibility of extension

## Results from syndromic surveillance of Acute Respiratory Infections (ARI) and Severe ARI (SARI) in Spain, seasons 2021-22 to 2023-24

**Figure S1. Weekly incidence of Acute Respiratory infections (ARI) attended in primary care, by age group, SiVIRA, seasons 2021-22 to 2023-24**

**
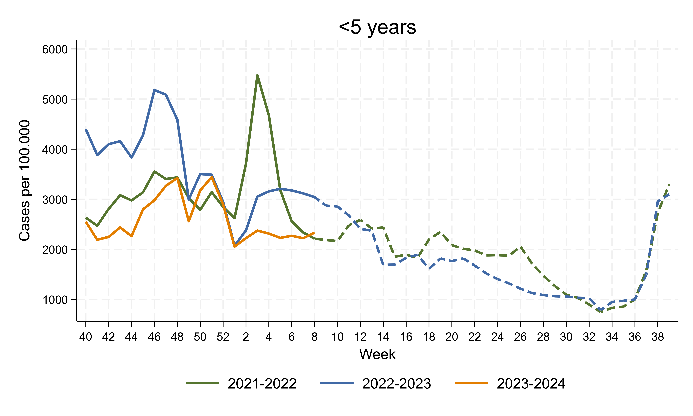

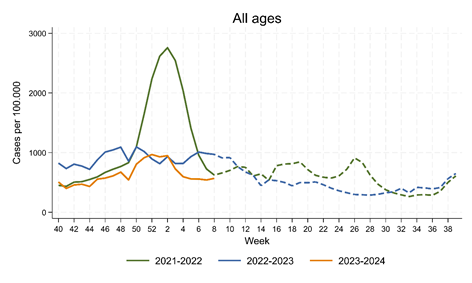
**

**Figure S2. Weekly incidence of Severe Acute Respiratory infections (SARI) in hospitals, by age group, SiVIRA, seasons 2021-22 to 2023-24**


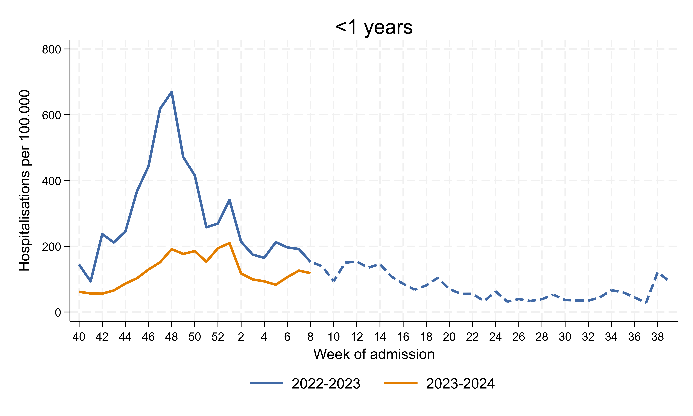

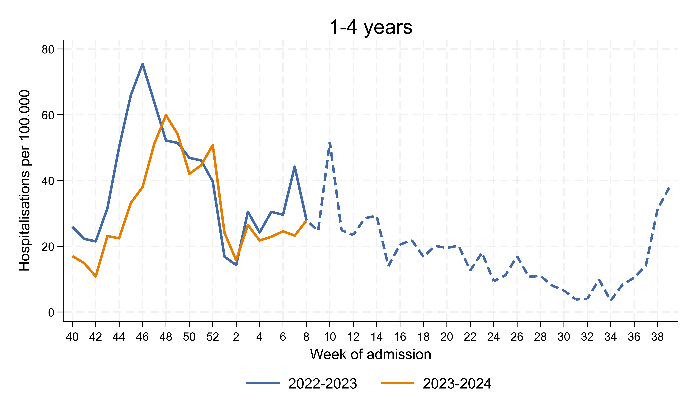

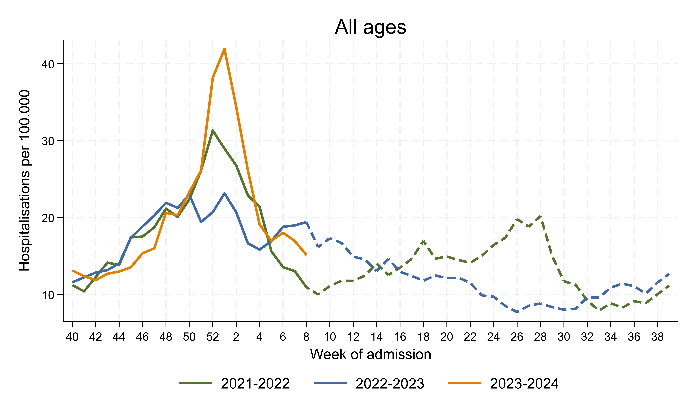


##

## Respiratory Syncytial Virus (RSV) positivity and incidence of RSV infections medically attended in primary healthcare in Spain, seasons 2022-23 and 2023-24

**Table S2. Number of ARI and SARI patients tested for RSV and number and proportion of RSV positives, by age group and season, SiVIRA, seasons 2022-23 and 2023-24**

| **Age group (years)** | **RSV positivity in ARI patients** | | | | | | **Age group (years)** | **RSV positivity in SARI patients** | | | | | |
| --- | --- | --- | --- | --- | --- | --- | --- | --- | --- | --- | --- | --- | --- |
|  | **40/2022 - 8/2023** | | | **40/2023 - 8/2024** | | |  | **40/2022 - 8/2023** | | | **40/2023 - 8/2024** | | |
|  | **Tests** | **Positives** | **%** | **Tests** | **Positives** | **%** |  | **Tests** | **Positives** | **%** | **Tests** | **Positives** | **%** |
|  |  |  |  |  |  |  | **<1** | 566 | 374 | 66,1 | 702 | 273 | 38,9 |
| **<5** | 2526 | 509 | 20,2 | 2225 | 458 | 20,6 | **1-4** | 409 | 148 | 36,2 | 464 | 206 | 44,4 |
| **5-14** | 2627 | 180 | 6,9 | 2117 | 126 | 6,0 | **5-14** | 131 | 21 | 16,0 | 212 | 25 | 11,8 |
| **15-44** | 5696 | 306 | 5,4 | 4831 | 171 | 3,5 | **15-44** | 141 | 10 | 7,1 | 342 | 16 | 4,7 |
| **45-64** | 4536 | 397 | 8,8 | 4282 | 193 | 4,5 | **45-64** | 519 | 36 | 6,9 | 1169 | 71 | 6,1 |
| **≥65** | 3446 | 345 | 10,0 | 3108 | 205 | 6,6 | **65-79** | 943 | 86 | 9,1 | 2128 | 170 | 8,0 |
|  |  |  |  |  |  |  | **≥80** | 1438 | 172 | 12,0 | 2740 | 277 | 10,1 |
| **All ages** | 18831 | 1737 | 9,2 | 16563 | 1153 | 7,0 | **All ages** | 4147 | 847 | 20,4 | 7757 | 1038 | 13,4 |

**Figure S3. Weekly incidence of RSV infections* in primary care by age group, SiVIRA, seasons 2021-22 to 2023-24**


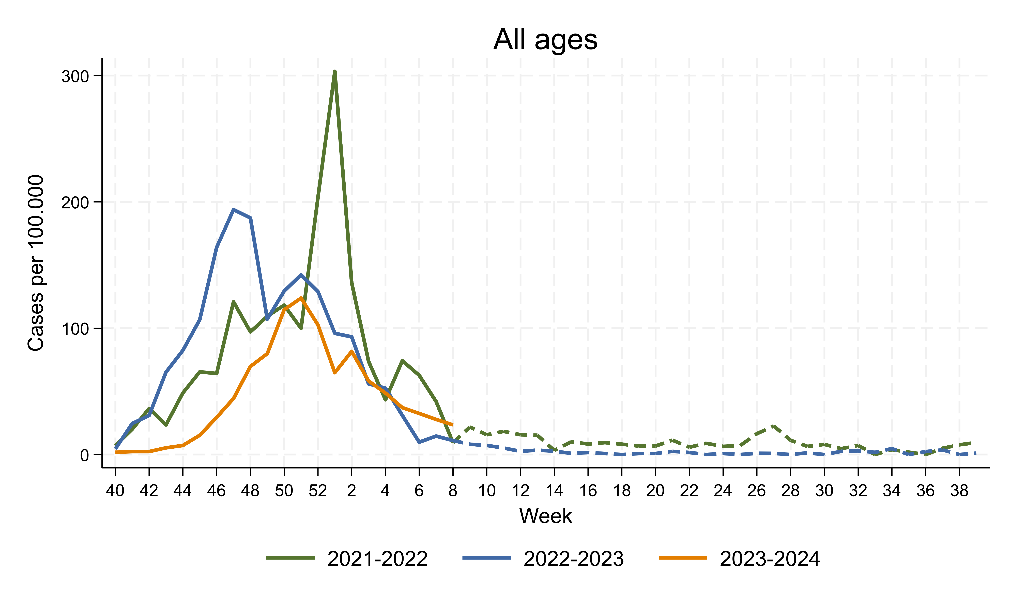


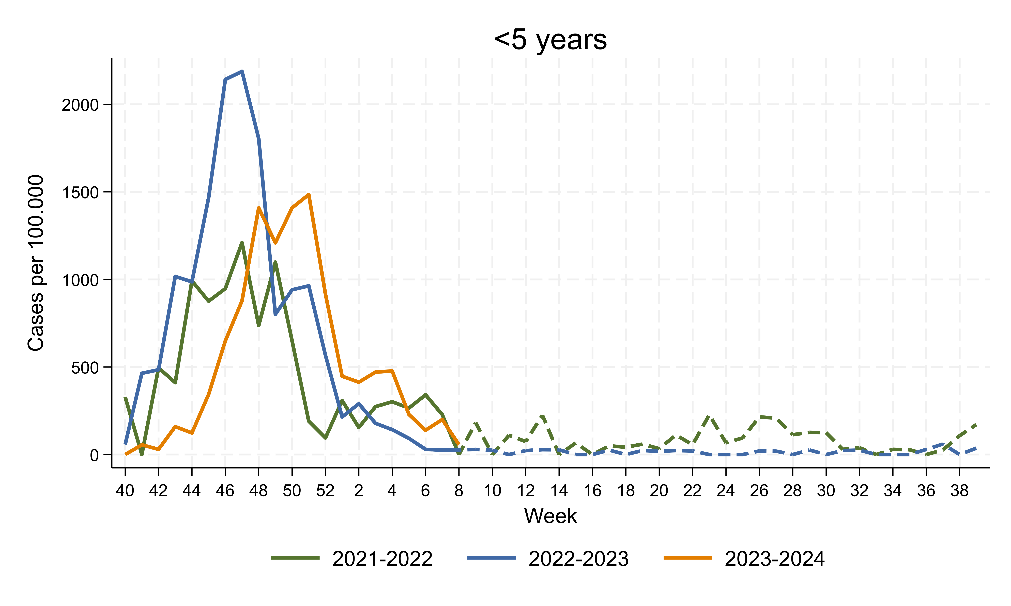


* The incidence of RSV infections*** is obtained by multiplying the ARI syndromic rates by the proportion of laboratory tests positive for RSV among a representative sample of those ARI that are systematically swabbed and tested.

**Table S3. Age distribution of confirmed RSV infections in primary care and RSV hospitalisations, SiVIRA, seasons 2022-23 and 2023-24.**

| **Age group (years)** | **RSV cases in primary care** | | | | **Age group (years)** | **RSV hospitalizations** | | | |
| --- | --- | --- | --- | --- | --- | --- | --- | --- | --- |
|  | **40/2022 - 8/2023** | | **40/2023 - 8/2024** | |  | **40/2022 - 8/2023** | | **40/2023 - 8/2024** | |
|  | **n** | **%** | **n** | **%** |  | **n** | **%** | **n** | **%** |
|  |  |  |  |  | **<1** | 13120 | 40,0 | 3357 | 14,5 |
| **<5** | 274725 | 32,4 | 199874 | 41,2 | **1-4** | 4494 | 13,7 | 4357 | 18,8 |
| **5-14** | 108569 | 12,8 | 68398 | 14,1 | **5-14** | 734 | 2,2 | 606 | 2,6 |
| **15-44** | 137833 | 16,2 | 72460 | 14,9 | **15-44** | 855 | 2,6 | 385 | 1,7 |
| **45-64** | 164978 | 19,4 | 55105 | 11,4 | **45-64** | 1664 | 5,1 | 1847 | 8,0 |
| **≥65** | 162323 | 19,1 | 89483 | 18,4 | **65-79** | 4351 | 13,3 | 4956 | 21,3 |
|  |  |  |  |  | **≥80** | 7589 | 23,1 | 7704 | 33,2 |
| **All ages** | 848428 | 100 | 485320 | 100 | **All ages** | 32808 | 100 | 23213 | 100 |
